# Automated Data Generation for Raman Spectroscopy Calibrations in Multi-Parallel Mini Bioreactors

**DOI:** 10.3390/s22093397

**Published:** 2022-04-28

**Authors:** Alexander Graf, Angus Woodhams, Michael Nelson, Douglas D. Richardson, Steven M. Short, Mark Brower, Marek Hoehse

**Affiliations:** 1 Sartorius Stedim Biotech GmbH, August-Spindler-Straße 11, 37079 Goettingen, Germany; alexander.graf@sartorius.com; 2 Sartorius Stedim TAP, York Way, Royston SG8 5WY, UK; angus.woodhams@sartorius.com; 3 Merck & Co., Inc., 2000 Galloping Hill Rd., Kenilworth, NJ 07033, USA; michael.nelson@merck.com (M.N.); douglas.richardson14@merck.com (D.D.R.); steven.short@merck.com (S.M.S.); mark_brower@merck.com (M.B.)

**Keywords:** Raman spectroscopy, mini bioreactor, process development, mammalian CHO cell culture, process analytical technology (PAT)

## Abstract

Raman spectroscopy is an analytical technology for the simultaneous measurement of important process parameters, such as concentrations of nutrients, metabolites, and product titer in mammalian cell culture. The majority of published Raman studies have concentrated on using the technique for the monitoring and control of bioreactors at pilot and manufacturing scales. This research presents a novel approach to generating Raman models using a high-throughput 250 mL mini bioreactor system with the following two integrated analysis modules: a prototype flow cell enabling on-line Raman measurements and a bioanalyzer to generate reference measurements without a significant time-shift, compared to the corresponding Raman measurement. Therefore, spectral variations could directly be correlated with the actual analyte concentrations to build reliable models. Using a design of experiments (DoE) approach and additional spiked samples, the optimized workflow resulted in robust Raman models for glucose, lactate, glutamine, glutamate and titer in Chinese hamster ovary (CHO) cell cultures producing monoclonal antibodies (mAb). The setup presented in this paper enables the generation of reliable Raman models that can be deployed to predict analyte concentrations, thereby facilitating real-time monitoring and control of biologics manufacturing.

## 1. Introduction

Protein-based biologics are becoming increasingly complex in terms of molecular attributes and the cell culture process required to manufacture them. A number of process development and technical transfer stages are required to optimize the product titer and critical quality attributes (CQAs) of biotherapeutics, before they can be delivered to patients.

Automated mini bioreactor systems have proven their potential for process optimization and development in several studies, as they are designed to have similar geometry to those found in pilot and commercial scale bioreactors [1]. These platforms have also been shown in scale-up studies to generate comparable cell growth and protein titer profiles up to the 3000 L scale [2,3]. Furthermore, the technology has been proven via several studies to have comparable performance to benchtop bioreactors [3,4,5] and has also been shown to be a qualified scale-down model (SDM) for commercial scale mAb production [6]. Thus, the process development is made much faster and, in turn, significantly shortens the time to clinic and consequently time to market.

Critical process parameters (CPPs), such as nutrient and metabolite concentration and protein titer in the cell culture, are mostly detected off-line, requiring regular sampling of the bioreactor. The need to routinely sample the bioreactor, which is typically restricted to trained laboratory staff, is both time- and labor-intensive. Additionally, the risk of bioreactor contamination during sampling is a common concern [7]. Finally, off-line sampling typically occurs at 24-h testing intervals, rendering direct process control of the measured analytes within relatively tight action limits not possible.

Raman spectroscopy is a well-suited PAT tool to nondestructively measure cell culture analytes in-situ, using immersion probes or flow cells. This vibrational technique uses a laser to generate monochromatic light that will scatter when interacting with complex biological samples. The inelastic scattered light yields structural information regarding the covalent bonds of the interrogated molecules with high molecular specificity and robustness. Raman spectroscopy has been widely adopted in biomanufacturing as a multipurpose analytical technique [8] for real-time monitoring of cell culture performance parameters, such as glucose, glutamine, glutamate, lactate, viable cell density (VCD) and product titer [9,10,11,12]. Additionally, Craven et al. [13], Berry et al. [14] and Rowland-Jones and Jaques [15] have monitored glucose via on-line Raman measurement, and Matthews et al. [16] have used Raman lactate measurements with feedback control to maintain glucose and lactate, respectively, at constant concentrations in CHO cell cultures. A study by Berry et al. [17] exemplified that Raman spectroscopy can be used to maintain glucose at low concentration levels in CHO cell cultures, which in turn reduced glycation in the mAb product from ~9% to 4%.

In-line Raman spectroscopy is mainly used with pilot and manufacturing bioreactors, due to scale limitations of the spectroscopic devices. More recently, preliminary studies suggest that this technique has the potential to be integrated in micro bioreactors (15 mL working volume) via automated routines to simultaneously measure multiple metabolites [15,18]. Presently, the largest barrier to the proof of this concept is the lack of an integrated reference system (e.g., glucose measurement used to construct the Raman calibration model), which in turn requires the use of off-line referencing systems that introduce model error as a result of the mismatch between the time of spectral acquisition and the reference measurement. Therefore, mini bioreactors featuring a robust, scalable, and fully integrated sampling and spectroscopic platform with automated solutions for liquid management, data acquisition, data alignment, and data export offer significant benefits. This minimizes manual intervention, de-risks bioreactor contamination, and mitigates the extensive work to compile calibration datasets that can otherwise be read by multivariate data analytics software. With this approach, a cost-efficient spectroscopy implementation is combined with the benefits of automation and data management, resulting in an efficient system for data generation and model building.

The creation of a robust Raman spectroscopy model that is applicable to production scales relies on the deployment of calibration models that correlate spectral signals with analytical measurements. As reviewed by Tulsyan et al. [19], constructing a quantitative model involves several critical steps. Initially, well-characterized spectral and analytical datasets are collected for cell culture parameters of interest, either from experiments in small-scale (benchtop) bioreactors or larger scale production bioreactors. Data pre-processing is subsequently performed to improve the signal-to-noise ratio and/or to reduce disturbances and to minimize equipment variations from the probe heads or spectrometers. In the penultimate step, these data are modeled using multivariate statistical methods, such as orthogonal partial least squares (OPLS), to correlate the spectral data to the parameter(s) of interest. Finally, the model is utilized for monitoring and control applications.

Good scientific practice for robust Raman model building relies on testing multiple cell culture parameters, ideally utilizing a design of experiments (DoE) approach, to obtain statistically relevant data. This includes challenging the model using independent datasets (i.e., those not used to construct the model) to determine if unknown correlations or trends are compromising performance. This is of particular importance for upstream bioprocesses, as nearly all analyte trends correlate with other analytes or with batch maturity. Ideally, this is accomplished by varying the process analyte conditions, such that the analyte targets fluctuate independently from one another. However, this may not always be practical and can be augmented with spiking studies, where pure analytes of interest are added to the bioreactor to build in the required spectroscopic dynamic range. The latter results in step changes of single analyte trends only being valid for the spiked analyte alone. Thus, producing a well-fitted, predictive Raman spectroscopy model can be time-consuming and costly in terms of the required media, reagents and staff resources, which ultimately can have a deleterious impact on commercialization timelines for biologics, such as monoclonal antibodies (mAbs).

In a typical example of a standard workflow for Raman model building, one study ran 37 separate production runs of a fed-batch bioprocess, ranging from a 2 L shake flask up to a 5000 L bioreactor [11]. They acquired measurements at 12 different (time) points across each run for a total of 444 data points to construct their Raman model. This kind of study is not only time-consuming but is also very expensive, particularly at larger scales (media costs alone are around €100,000 for a 2000 L run). Furthermore, executing model building runs in production environments is almost impossible, as induced process variations would require the batch to be discarded due to suboptimal quality attributes for the end biological product. Therefore, a robust Raman model is currently achieved by monitoring dozens of production runs and arbitrary (smaller) variations of in-specification batches, with considerable value being introduced when unfortunate out-of-specification events occur during routine manufacturing.

Utilizing a small-scale bioreactor setup enables the use of sound experimental design methodologies to induce variations at acceptable costs, while one run consisting of twenty-four bioreactors yields a larger and more robust design space compared to many more large-scale production batches, consisting of only arbitrary variations. If this model can be transferred to larger scales (e.g., by adding a few points from larger scales), the robustness of the low effort small-scale bioreactor model is made available for applications across all scales.

This study describes the implementation of a prototype Raman flow cell in a 250 mL mini bioreactor system, ultimately paving the way to monitor and control key process and product attributes during small-scale cell culture manufacture. The system described herein enables automated bioreactor sampling and reference measurements paired with on-line Raman spectroscopy, which facilitates robust model building through the analysis of induced process variations, according to DoE principles and automated spiking experiments. The prototype Raman spectroscopy probe head connected to the flow cell utilizes an optical interface that can also be found in single-use bioreactors at 50 to 2000 L scale, which facilitates the scalability of the Raman model. The application of this PAT tool across the scales has the potential to rapidly deliver high quality data during process development, which could later be applied to commercial manufacturing for on-line monitoring and control of large-scale bioreactors.

## 2. Materials and Methods

### 2.1. Experimental Setup, Raman Spectroscopy Integration Prototype and Reference Measurements

**Figure 1:**
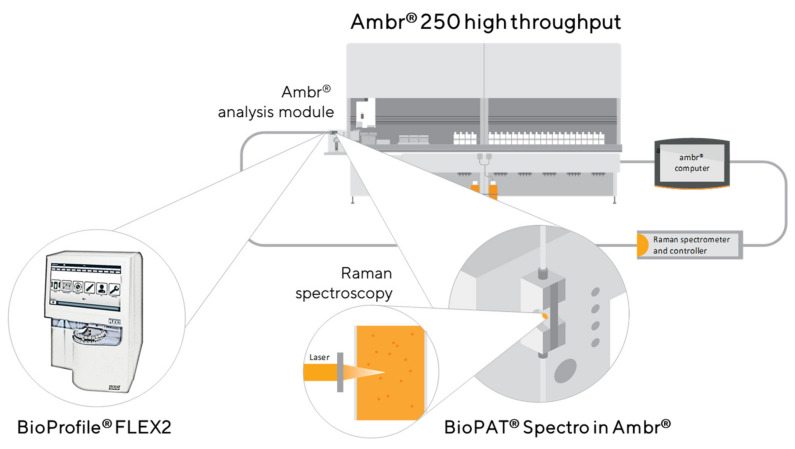
Experimental setup: schematic diagram of the automated 250 mL mini bioreactor system (Ambr^®^ 250 high throughput, Sartorius, Royston, UK) with a prototype Raman flow cell (path length 1 mm; internal volume ~40 µL) integrated into the Ambr^®^ analysis module, enabling automated Raman spectroscopy and an integrated reference measurement system (BioProfile FLEX2, Nova Biomedical Corporation, Waltham, MA, USA).

An automated 250 mL bioreactor system with 24 vessels (Ambr^®^ 250 High Throughput, Sartorius, Royston, UK) was equipped with an integrated analysis module, including a prototype Raman flow cell, to enable automated spectroscopic Raman measurements and with an integrated cell culture analyzer to enable reference measurements. The liquid handling capability of the analysis module was extended by adding a prototype optical flow cell (1 mm path length, sapphire windows, ~40 µL volume) (Figure 1). The entrance to the flow cell was connected to the sample cup, while the exit port was diverted to a waste bottle.

Culture samples from the mini bioreactors were analyzed using an integrated Bioprofile^®^ FLEX2 (Nova Biomedical Corporation, Waltham, MA, USA) automated cell culture analyzer with an External Sample Module (ESM), which managed and transferred samples (0.5 mL) for pH, pCO_2_, pO_2_, VCD, glucose, glutamine, glutamate, lactate, and ammonium analysis.

Titer reference data were generated upon the completion of the experiment, by analyzing refrigerated cell-free bioreactor samples from select process days via Protein A affinity high performance liquid chromatography (ProA HPLC).

A prototype optical probe head was connected to the flow cell and HyperFlux PRO Raman Spectroscopy system (Tornado Spectral Systems, Toronto, ON, Canada). The latter was also connected to an Ambr^®^ 250 system for the automated generation of Raman spectra. The Ambr^®^ 250 software was modified to allow instrument control of the Raman spectrometer (start/stop measurement) and the transfer of spectral data from the spectrometer to the bioreactor control software. These data were then merged with relevant bioreactor data (e.g., vessel number, sample ID, batch ID, sampling time, batch age, and reference data) and jointly exported as a CSV file for statistical analysis and model building.

### 2.2. Cell Lines and Media

A Chinese hamster ovary (CHO) cell line expressing an IgG4 monoclonal antibody (mAb) was used for the cell culture experiments. The cells were expanded and subsequently cultured in proprietary, chemically defined basal media. Starting from a cryo-vial thaw, the cells were serial passaged into consecutively larger shake flask cultures, until a desired cell count was achieved to inoculate the 250 mL bioreactors at the specified seeding densities.

### 2.3. Cell Culture and Data Acquisition Process

To produce statistically relevant data for robust Raman Spectroscopy model building, a run was performed in the Ambr^®^ 250 high throughput mini bioreactor system (Sartorius, Royston, UK), utilizing 24 mini bioreactors at a working volume of 180–250 mL. As for the standard settings, the bioreactors were operated at a 36.5 °C, 400 rpm stirring speed, and a dissolved oxygen (DO) of 30% air saturation for 14 days. The pH was controlled between 6.8 and 7.1, with CO_2_ sparging to decrease the pH and the addition of an NaOH base to increase the pH as needed. Proprietary, chemically defined feed media were added throughout the run, based on the daily metabolite readings in the reactors. The feed regimen was based on a combination of continuous and bolus feeds, as described in the work by Manahan et al. [6].

**Figure 2:**
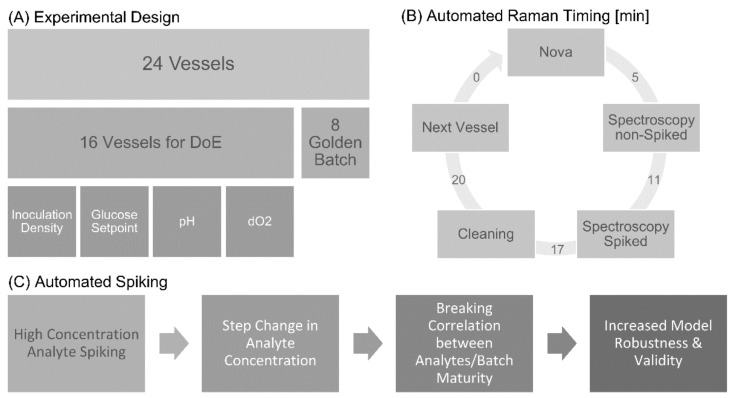
Experimental design and data acquisition of Ambr^®^ 250 runs to generate Raman spectra and reference analyte data. (**A**) In 16 DoE vessels, inoculation density, as well as glucose, pH and dissolved oxygen setpoint were varied, while process settings were kept constant in the 8 Golden Batch vessels, which served as control replicates. (**B**) Schematic diagram illustrating automated data acquisition and duration. Notably, the time gap between the FLEX2 reference measurement and Raman spectroscopy of the non-spiked sample is only 5 min. (**C**) Schematic diagram illustrating that automated spiking leads to increased model robustness and validity.

For the process run, a design of experiments (DoE), analogous to the one that was used during standard commercial process development, was replicated for this case study (Figure 2A). The process parameters of initial cell density, daily glucose feed target, pH setpoint, and DO setpoint were controlled and deliberately varied in sixteen vessels, while the remaining eight vessels were kept at the standard process settings mentioned before for control replicates. Automated reference measurements were carried out using an integrated Bioprofile^®^ FLEX2 as previously described. This experimental setup was chosen in order to demonstrate that Raman data generation can be accomplished in tandem with a standard commercial process development experiment, to take advantage of the wide range of metabolite and product titer profiles generated from the experimental design. In general, the presented approach is also feasible in other experimental set-ups, such as OFAT trials. However, this may result in lower variations within some of the analytes and lead to inferior model performance or the need of additional runs.

Sequentially, during this high-throughput study, Raman spectra were periodically acquired from bioreactor samples both before and after being spiked (spiking from day six onwards), with known concentrations of key analytes (Figure 2B,C; Table S1). A total of 48 samples (24 unspiked and 24 spiked) were collected on a daily cadence and analyzed via Raman spectroscopy. All the spectroscopy related liquid handling was automated and performed overnight to not interfere with the standard daily sampling and control, as well as feeding. For spiking, one sample per vessel (140 µL) was taken and mixed with one volume of one of the following analytes: glucose, lactate, glutamine, or glutamate (nominal spiking volume 20–60 µL of analyte stock solution), as well as purified “mAb1” protein product (generated from previous experiments), according to the scheme shown in Table S1. The spiked samples were mixed in a microwell plate directly before analysis via Raman spectroscopy. As the spiking solution is composed of a different matrix to the bioprocess sample, the addition of high amounts of the stock solution may lead to a significant change in the spectrum compared to the unspiked sample and in turn, lead to more outliers or offsets, but neither were observed (Figure S1A) in Supplementary Materials. Two stock solutions, one lower and one higher in concentration, were prepared for each analyte to generate an equally distributed range. The precise concentrations of stock solutions varied for each analyte, depending on the normal process range between 4 and 32 g/L. Spiking studies were used to break the correlations between the different analytes of interest, as well as with batch maturity. Since the spiking of one analyte at a time leads to the decrease in all the other analytes, this could possibly introduce new correlations. To minimize this effect, high stock solutions as well as different combinations of stock concentration and spiking volumes were utilized. As previously reported by Rowland-Jones, Graf et al., the positive effects of breaking correlations are far stronger than any newly introduced ones [18].

The sampling scheme consisted of one reference measurement per vessel, followed by the acquisition of spectra from an unspiked sample, and finally from the spiked sample, as shown in Figure 2B.

The FLEX2 reference measurements were used to align with spectra from the unspiked samples. The concentrations of the spiked samples were automatically calculated by the Ambr^®^ software, using both the reference value of the unspiked sample combined with the concentration and volume of the added stock solution. To minimize errors that stem from the liquid handling system, the systematic offset of the pipetting at the relevant volumes was determined in advance and used for these calculations (Table S1). If necessary, different amounts of air in the sample, e.g., due to foaming, could be programmed into the liquid handling system. Within this study, foaming was not a source of disturbance, just as changing viscosities within the samples, e.g., due to increasing cell counts.

The reference measurements for the mAb1 protein titers were more limited, since the integrated FLEX2 had no protein titer assay. Therefore, an additional unspiked set of samples from each bioreactor was taken on every second day over the duration of the cultivation and one further set of spiking samples just on day four. For this analyte, the spectra were captured directly after sampling automatically, similar to before, while all titer reference data were generated upon the completion of the experiment, by analyzing the refrigerated cell-free bioreactor samples via ProA HPLC. To align the spectra and reference values, the latter were first manually normalized to the highest value and then matched by vessel number and timepoint to the corresponding spectrum within SIMCA 16 (Sartorius Data Analytics AB, Umea, Sweden).

Overall, the single run produced a theoretical total of 528 Raman spectra from 24 vessels measured daily without spiking and including the additional spectra of the spiking samples from day six onwards.

### 2.4. Raman Spectroscopy Measurement

All measurements were acquired using a HyperFlux PRO Raman Spectroscopy system (Tornado Spectral Systems, Toronto, ON, Canada), equipped with a 785 nm laser for excitation set at 495 mW. As previously described, the system was controlled by the Ambr software. Here, the overall measurement time per sample was set to 5 min, which was comprised of an average of five separate 1 min spectra taken from one sample (calculation within the Ambr software (BioPAT Spectroscopy Data Manager, Sartorius, Royston, UK)). Each 1 min spectrum was, in turn, averaged from *x* spectra measured at a set integration/exposure time *I_t_* within the spectrometer control software. The increase in fluorescence throughout the run required a reduction in *I_t_* to not risk sensor saturation. Thus, the averaging (*avg*) was adapted by the operator accordingly, to guarantee an acquisition time of 1 min in all cases (e.g., *I_t_ =* 0.2 s, *avg* = 300; *I_t_ =* 1 s, *avg* = 60). Maintaining the overall acquisition time similar ensures that the signal to noise ratio within each spectrum stays identical; therefore, the spectrum can be recalculated to the initially longer exposure time without a loss of sensitivity. The design of the measurement chamber was optimized to block any direct incoming ambient light; however, a dark scan was performed prior to each sample measurement to mitigate the impact of any variations in ambient light that might have strayed into the sample chamber.

### 2.5. Multivariate Raman Model Construction

Orthogonal partial least squares (OPLS) regression models were developed from acquired Raman spectra which were correlated to standard reference measurements performed using the integrated FLEX2 bioanalyzer or the ProA HPLC for the mAb titer. Spectral and reference data were first aligned within the Ambr^®^ software in an automated fashion, followed by averaging five spectra from each sample (each spectrum having a total acquisition time of one minute) to improve the signal-to-noise ratio.

Initially, before data pre-treatment, the raw spectra were scanned to exclude aberrant measurements (e.g., due to an empty measurement chamber), by checking the overall intensity and that of the water bands in particular. Due to the different levels of fluorescence background, all spectra were then baseline-corrected with an asymmetric least-squares algorithm [20] (Figure S2B), as this correction method proved to be superior to the standard pre-treatment with standard normal variate (SNV) and first derivative analysis. The water band at 1650 cm^−1^ was utilized to normalize all spectra, and therefore correct the potential variations that were not caused by the process itself, but to other confounding factors (Figure S2C). Examples for these disturbances are small air bubbles still present in the measurement chamber, or differences in the optical sampling volumes, due to varying cell densities. As described in several studies, the Raman water signal can be exploited for this task very reliably [10,18,21]. As the sample mainly consists of water, small peaks from other analytes that may be present in this region can be neglected. All pre-treatments were done in Python 3 with the help of the numpy, pandas, and rampy module and similar modules for these pre-treatments can also be found in R.

After pre-treatment, the spectra and reference data were transferred to SIMCA 16 Multivariate Data Analysis software (Sartorius Data Analytics AB, Umea, Sweden). A PCA model was built to identify the possible outliers outside the Hoteling’s T^2^-boundaries in the score plot, which might have been missed in the previously mentioned investigation. Any remaining outliers were identified at a later stage of model building, using the observed vs. predicted plot after verification with their distance to the model (DmodX) value. While a completely empty measurement chamber is easy to detect, these additional outliers can stem from several minor reasons. These causes include sub-optimal sample transfer into the measurement chamber and, therefore, some amount of air or tiny air bubbles is still present, which obscures the measurement. Depending on the ratio and position of the air in relation to the laser focus, in some cases, this can be corrected by the water band normalization. Other reasons for outliers can be miss-measurements of reference analytics, or falsely calculated analyte concentrations after spiking, due to bad mixing of the sample. In summary, the overall number of outliers stayed well below 5%.

Quantitative models for the different analytes of interest were generated with the help of an OPLS algorithm. The X-block consisted of the spectral variables that were mean-centered, while the Y-block was composed of the scaled (unit variance) reference measurements. OPLS, instead of classic PLS, was chosen, as it increases the model interpretability while maintaining the same predictive power. This increase is achieved by removing variance in the X-Block (i.e., the spectra), which has no correlation to the variation in the Y-Block (i.e., the reference data) or in mathematical terms, removing systematic variation in X that is orthogonal to Y. This results in a model that consists of one predictive component (as one model is built for each Y-variable separately) and a number of orthogonal components that can differ in each model [22,23]. Each model is, thus, denoted by 1 + *x* principal components. To further reduce the impact of the correlations between the analyte trends and batch maturity, the spectral regions for each analyte were matched to those that were found to be unique to the analyte of interest in previous trials [18] (Table S2). In this case, DoE studies with mixtures of the main analytes were performed to determine the unique spectral regions of interest for each analyte. Additionally, these lab trials using a simplified system (accurate concentrations in buffer/water) are good indicators of the maximum achievable performance of the technique. Lower prediction errors in cell culture than in the lab DoE are an indicator for in-process correlations that help to decrease the prediction error; however, models with this prediction error profile bear the risk of model failure, as future changes to the process may invalidate these correlations that the model is highly dependent on. Even though model building relies on the user’s experience, especially when it comes to overfitting, certain (statistical) tools can be used as a basis for selecting an optimal model. The goodness of fit (R^2^) and goodness of prediction (Q^2^) should be as high as possible, while simultaneously minimizing the difference between them. Differences larger than 0.3 can indicate either the presence of outliers in the dataset, which may have sufficient justification to be omitted from the dataset, or that the model is overfit, requiring one or more model components to be removed in order to enhance accuracy and long-term robustness [23].

Several performance metrics, such as the root mean square error evaluation (RMSEE) and the root mean square error of cross-validation (RMSEcv), are essential markers for the estimation of model error. While the RMSEE shows how well the model performs, the RMSEcv indicates how well the model can predict future datapoints, given that this data does not deviate significantly from the original dataset. Both of these error values should be as low as possible, while simultaneously minimizing the difference between them, as larger divergences between the two metrics indicates the overfitting of the model to the available data. The two values are calculated as follows, with *n* being the number of samples in the model, *y_i_* denoting the observed reference value, and *y_cal_*, and *y_CVpred_*, representing the predicted values from the model and cross-validation, respectively:

(1) $$\mathrm{RMSEE}=\sqrt{\sum \frac{{\left(y_{i}-y_{cal}\right)}^{2}}{n}}$$

(2) $$\mathrm{RMSEcv}=\sqrt{\sum \frac{{\left(y_{i}-y_{CVpred}\right)}^{2}}{n}}$$

The cross-validation (CV) groups must be consciously selected for the RMSEcv to be a reliable measure of model performance. For example, the selection of a high number of CV groups results in a lower number of samples that are left out of testing during the cross-validation routine, ultimately serving as a weaker challenge to the model under consideration. The reader is directed to a study by Eriksson et al. [23] for additional information on how cross-validation works and its associated advantages and limitations.

Consequently, the trial dataset was split into four CV groups according to their vessel number. Therefore, complete batches were left out of the sub-models of the cross-validation routine. The selection of four CV groups was determined to be a good compromise between omitting too many samples (leading to highly inaccurate CV models) and leaving out too few samples (therefore not stressing the model enough) to obtain dependable RMSEcv values. Additionally, cross-validation with full batches can also be understood as an average of four external datasets yielding a better overview of the model performance when challenged with fully independent datasets, as opposed to using a single external test set with 75% of batches and predicting the remaining 25% of vessels.

## 3. Results and Discussion

### 3.1. Data Generation with the Experimental Prototype

For a fully automated sample analysis, an Ambr^®^ 250 was modified to integrate both a FLEX2 metabolite analyzer and a HyperFlux PRO Raman Spectroscopy system, equipped with a second-generation prototype probe head and a novel micro-volume flow cell (Figure 1). The analyte concentrations, measured via the integrated FLEX2, were used as the reference points for the development of the predictive Raman model. Multiple actions were required to complete the analysis of the cell culture samples, including (i) the withdrawal of a sample from the mini bioreactor via the automated liquid handler, (ii) the distribution of the sample to the FLEX2 analysis module via the External Sample Module (ESM), (iii) the release of the sample into the analysis module (AM) sample cup, (iv) the discard of the residual sample into the ESM waste, and (v) a cleaning cycle with standard washing liquids to prevent sample carry over.

Antibody titer reference measurements were generated via an off-line ProA HPLC analysis of the clarified daily sample retains.

The wavelength axis of the spectroscopy instrument was first calibrated with a mercury-argon lamp (HG-1, Ocean Optics, Orlando, FL, USA). The white light spectrometer calibration (also referred to as y-calibration or intensity calibration) included the complete optical path by back illumination via a separate fiber connection of the probe head, connected to a tungsten halogen light source (HL-2000, Ocean Optics, Orlando, FL, USA). Reference samples were automatically taken and measured by the integrated FLEX2 system immediately prior to the Raman measurement. The analysis of a cell culture sample from each mini bioreactor for Raman measurements involved the liquid handler withdrawing and releasing the sample into the AM sample cup. With the help of an integrated syringe pump, the sample was transferred to a flow cell for Raman measurements. To prevent sample carryover, the sample was discarded following spectral analysis and a cleaning cycle was initiated with standard AM washing liquids.

The spiked samples required manual manipulation by the robotic liquid handler. First, a cell culture sample (~250 µL) was dispensed into a microwell plate. Then, a certain volume of stock solution was aspirated in a tip, followed by the aspiration of 140 µm of the cell culture sample in the same tip. Next, the cell culture sample and stock solution were mixed in a different well by pipette aspiration and release, and finally transferred to the sample cup for delivery to the flow cell for spectral analysis.

The acquired spectra from the unspiked samples were time-aligned with FLEX2 measurements to merge the two datasets. The concentration of spiked samples was automatically calculated in the Ambr^®^ system software, using the reference value of the unspiked sample and accounting for the spike itself (i.e., volume addition and stock solution concentration). The careful integration of the software and hardware components yields negligible time differences between the reference and spectral measurements, which ultimately increases the accuracy and selectivity of the models compared an application capable of using only off-line reference measurements (e.g., those acquired via a reference analyzer that may or may not reside in the same physical location as the spectrometer).

One single run of the experimental setup described in this paper produced a total of 528 Raman spectra from 24 vessels (16 DoE and 8 Golden Batch vessels) measured twice a day (same sample unspiked and spiked) for 14 days. Those spectra, together with the simultaneously acquired reference data, were then used to develop the predictive OPLS models.

### 3.2. Predictive OPLS Models

**Figure 3:**
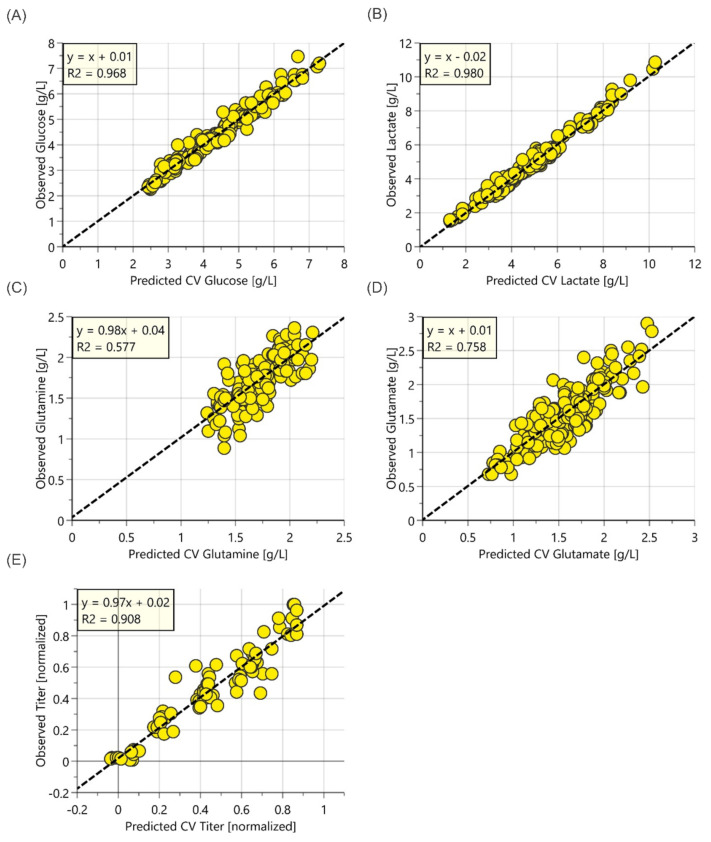
Parity plots of the observed values vs. predicted values from cross-validation for OPLS models of unspiked cell culture samples for (**A**) glucose, (**B**) lactate, (**C**) glutamine, (**D**) glutamate and (**E**) normalized titer with unspiked cell culture samples from 250 mL mini bioreactors.

Individual OPLS models were developed to correlate the pre-processed Raman spectral data with the reference data to produce predictive models of glucose, lactate, glutamine, glutamate and mAb titer. Figure 3 shows the parity plots for these analytes using the measured reference values and the predictions during cross-validation from the Raman-based models.

Generally, high cell densities did not have an impact on either the liquid handling system nor the quality of spectra, i.e., resulting in more outliers. Peak viable cell densities of up to 14.5 × 10^6^ cells/mL were reached towards the middle of the cultivation. Whenever the viable cell density decreases by several million cells per mL towards the end of the run, the data points drift away from the main group (Figure S1B), indicating a fundamental change in the spectra. However, these data points are not necessarily outliers and are mostly still part of the analyte models shown below. In summary, this shows that (a) the liquid handling system works reliably at different viscosities, (b) high cell densities and, therefore, increased turbidity do not interfere with the spectroscopic measurement and (c) when fundamental changes within the process occur, the data pre-treatments are able to compensate these.

Table 1 summarizes the key parameters and figures of merit for the models constructed using unspiked bioreactor samples. The Raman models generated in this study showed lower prediction errors for glucose and lactate measurement than those published, e.g., by Rowland-Jones, van den Berg et al. [24], where the measured glucose concentration showed a RMSEcv of 0.92 g/L and lactate concentration showed a RMSEcv of 1.11 g/L in a similar process. The normalized product titer model also showed good accuracy, with a Q^2^ value of 0.911 and RMSEcv of 0.08 g/L.

The Raman models for the unspiked samples of glutamate and glutamine were found to have lower coefficients of determination, with a Q^2^ value of <0.8, indicating lower predictive ability. The main issue with the unspiked models is that glutamine and glutamate are present over a very narrow concentration range (1 g/L for glutamine and 2 g/L for glutamate), which makes it more difficult for the chemometrics algorithm to distinguish between the real concentration changes and process noise. Additionally, Raman scattering for lactate and glucose produces more dominant bands than for glutamine and glutamate. Bearing in mind the limitations, spiking might be useful to increase the analyte range to improve model quality. These observations are consistent with other cell culture studies where weaker Raman activity, particularly with glutamate [14,25,26], has produced less accurate models compared to those for glucose or lactate.

Altogether, the process at hand delivered comparably high concentrations of all the analytes without concentration levels below 0.5 g/L, except for the mAb titer. If the models are transferred to other scales or the processing is changed, possibly leading to lower concentrations than those covered in the model, this can result in higher prediction errors, or in the worst case, to a wrong prediction of a much higher analyte concentration than actually present. This, in turn, could leave the model useless for process monitoring and control. To counteract this, various measures can be taken to further extend the range of the model to cover lower concentrations. These include, instead of spiking, the dilution of the sample with an analyte of interest-free medium (i.e., glucose or glutamine free) and an added experiment where some vessels are either run in batch mode, or have a significantly different feeding regimen, thus leading to a deprivation of the metabolites. A third choice would be to combine data from this process with data from a different process that ideally uses similar media, but is run differently and is known to have lower analyte concentrations present. Previous studies have shown that the latter is possible, even if the cell lines and target products are different [18]. Overall, past studies have shown that the limits of detection for the different analytes between 0.1 g/L and 0.3 g/L with Raman spectroscopy in bioprocessing can be achieved [18,27].

### 3.3. Predictive OPLS Models of Combined Non-Spiked and Spiked Samples

**Figure 4:**
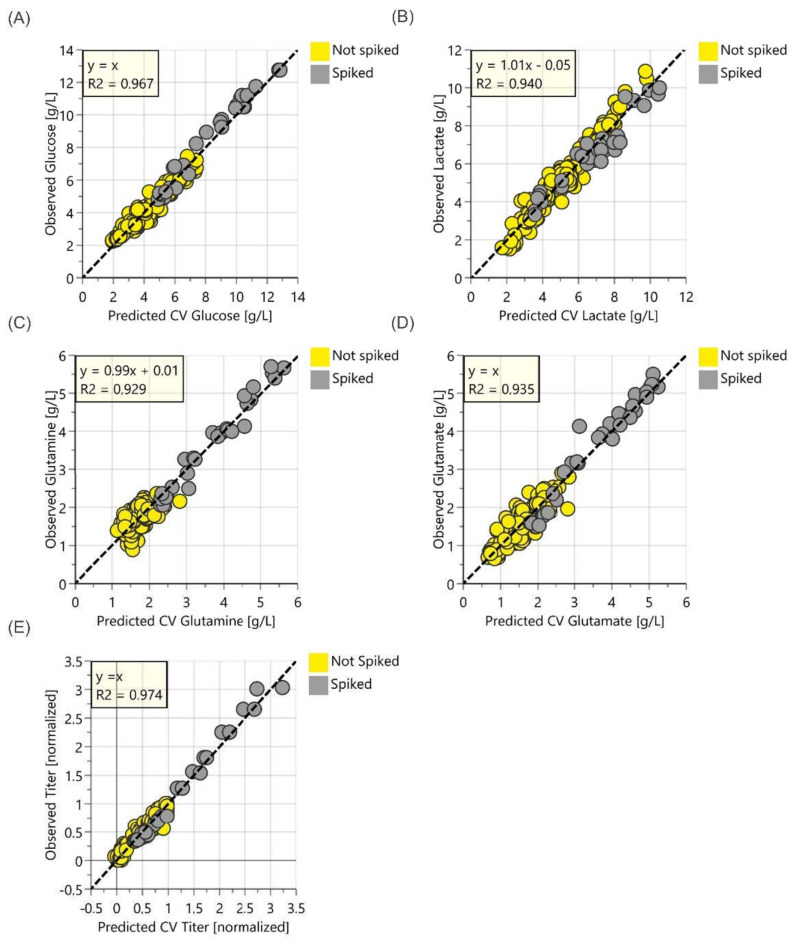
Parity plots of the observed values vs. predicted values from cross-validation for OPLS models, including spiked cell culture samples for (**A**) glucose, (**B**) lactate, (**C**) glutamine, (**D**) glutamate and (**E**) normalized titer with spiked cell culture samples from the 250 mL mini bioreactors.

OPLS models, including spiked data, were employed to extend the dynamic range and potentially improve the correlation with reference data. These models were developed using the same pre-processing and outlier identification methods as previously discussed for unspiked samples. Figure 4 shows the observed (reference) versus the predicted (Raman) data from the models developed for glucose, lactate, glutamine, glutamate and titer in spiked cell culture samples.

Additionally, not all the cell culture processes yield high concentrations of glutamine and glutamate (highest concentration e.g., below 1 g/L). For these processes, spiking can make the difference between a functional and a non-functional model. Spiking, therefore, has the potential to generate more valid OPLS models for glutamine and glutamate. Table 1 shows the summary statistics of the spiked and unspiked Raman OPLS models.

The models including both spiked and unspiked data were developed. Generally, the models for a particular analyte demonstrated good performance, regardless of whether spiked or native samples were used. However, the inclusion of spiked samples for glutamine and glutamate (extending the concentration range of the unspiked samples) resulted in a better fit, with a Q^2^ values above 0.9 and RMSECV values of 0.24 g/L and 0.23 g/L, respectively. This indicates that Raman models can predict glutamine and glutamate concentrations within a similar accuracy and specificity as previously demonstrated for other analytes (e.g., glucose, lactate), further instilling confidence that the predictive performance of the models for these less explored analytes is limited by spurious correlations.

While spiking did extend the calibration range for glucose and lactate models, it did not have a considerable positive impact on the Q^2^ values for either analyte (Table 1). This is a consequence of the variations in the process conditions via the DoE, the wavenumbers selected for inclusion in the models, and the large concentration range of lactate and glucose in the native samples, which together resulted in minimal residual variance that was unexplained by the optimized models. Additionally, the fed-batch process requires the addition of feed media, such as nutrients, that are naturally depleted during the course of cell culture, which enhances the glucose range as this was a component of the media used in this case study. It should be noted that the models based on spiked samples are expected to have a slightly higher model error than the optimal models based on non-spiked data. This anticipated decrease in model accuracy is a result of the variations caused by the slight inaccuracies associated with the stock solution concentration and additional pipetting and mixing steps, all of which impact the ability to successfully correlate the changes in Raman scattering, with the corresponding changes in reference values.

All the Raman generated models showed a slight difference in the slope/offset between the spiked (Figure 4) and unspiked (Figure 3) samples, which was most notable for glucose. The differences in slope and offset were hypothesized to be a consequence of sample dilution or insufficient mixing. To evaluate this hypothesis, further tests were performed, such as comparing several measurements taken one from one mixture, as well as comparing different mixing procedures. These tests proved non-optimal mixing of the sample and spiking solution as the sources of these variations. The pipette mixing technique of the early prototype led to the moving of layers, but to the insufficient mixing of these layers. This resulted in a concentration gradient between the first and the last part of the spiked sample. Given that the first portion of the sample was also used to rinse the flow cell, a Raman analysis was performed on the samples with concentrations that exceeded what nominally should be present when assuming sample homogeneity. This led to further refinement of the spiking methodology, which minimized the differences between the spiked and non-spiked samples, suggesting that sample inhomogeneity was effectively mitigated. The confirmation of the lab results is planned as a part of a separate study and will require separate cultivations, which can serve as independent prediction datasets to further evaluate the accuracy and robustness of the models discussed herein.

### 3.4. Application of Raman Models

**Figure 5:**
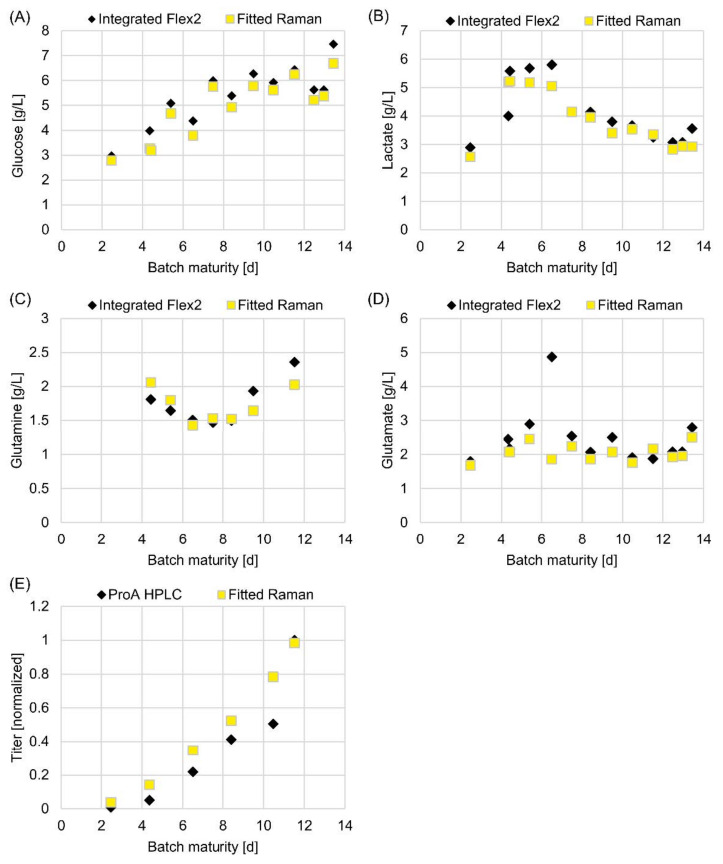
Observed vs. predicted development of analyte concentration during batch maturation. Comparison of observed (by reference method; black diamonds) and predicted (from the Raman spectroscopy models; yellow squares) analyte concentration vs. batch maturity from cell culture in a single 250 mL mini bioreactor; (**A**) glucose, (**B**) lactate, (**C**) glutamine, (**D**) glutamate, (**E**) mAb titer.

Robust and predictive OPLS models for glucose, lactose, glutamine, glutamate and mAb titer in cell culture were developed using Raman spectra, generated with the integrated 250 mL mini bioreactor system. To determine the potential of using spectral data for on-line monitoring, one specific 250 mL vessel was selected to illustrate the analyte levels over a 14-day process run. The data of this vessel were not used in model building, in order to validate the model performance with an outside dataset. The results (Figure 5) demonstrate that the Raman model for the selected analytes, as well as the mAb titer, align well with the reference data, indicating that the Raman models alone are likely to be suitable to control the cell culture. It is of particular note that the models even provide reasonable data for the glutamate concentration on day 6 and mAb titer on day 10, when the suspected sampling errors are believed to have caused reference measurement deviations. Overall, these results are promising and support the next phase of work, which is to transfer models based on small-scale data to larger scale single-use bioreactors to evaluate model scalability.

## 4. Conclusions

Mini bioreactor systems are increasing in popularity within the biopharmaceutical industry, fueled by the growing library of applications underscoring their ability to reduce timelines and the overall cost associated with process development and cell culture scale-up for manufacturing. A prototype system, integrating a Raman spectrometer into a multiparallel mini bioreactor system with an integrated bioanalyzer, was designed to facilitate the continuity of PAT across scales. A CHO cell line expressing a mAb was selected as a case study to demonstrate that accurate and robust Raman models could be developed using the prototype system, which would be difficult and costly to generate using conventional model building approaches that are currently predicated on larger bioreactors.

Notably, the integration of both a Raman flow cell and a bioanalyzer allowed for a reliable correlation between the Raman spectra and reference measurements because this experimental setup eliminates the otherwise necessary time-consuming manual collection of reference data. Due to the minimal time gap between the reference measurement and Raman spectroscopy, the models generated in this study show even lower prediction errors than the models generated with similar systems, but with manual collection of reference data [24].

In conclusion, using a multi-parallel mini bioreactor system integrating Raman spectroscopy and reference measurements, this study generated automated Raman spectroscopy models for a range of nutrients, metabolites and product titer, offering the potential to significantly reduce time and resource costs of commercial process development. Future case studies will investigate under which conditions the models generated in this study can be transferred to larger scales and whether they can be utilized for process monitoring and control.

## Figures and Tables

**Table 1:** Summary of key parameters for Raman OPLS models from spiked and unspiked samples from 250 mL mini bioreactors. Range indicates the concentration range of the analyte within the model; *n* states the number of datapoints used in the model; # of principal components states, in addition to one predictive component, how many orthogonal components the model utilizes; Q^2^ denotes the goodness of prediction; RMSEcv equals to the root mean square error of cross-validation.

Analyte	Range	*n*	# of Principal Components	Q^2^	RMSEcv
Glucose	2–8 g/L	224	1 + 1	0.97	0.20 g/L
With Spiking	2–13 g/L	254	1 + 3	0.97	0.36 g/L
Lactate	1.5–11 g/L	221	1 + 3	0.98	0.23 g/L
With Spiking	1.5–11 g/L	255	1 + 5	0.94	0.43 g/L
Glutamine	1–2.5 g/L	146	1 + 4	0.58	0.21 g/L
With Spiking	1–6 g/L	173	1 + 3	0.93	0.24 g/L
Glutamate	0.5–2.5 g/L	215	1 + 4	0.76	0.21 g/L
With Spiking	0.5–5.5 g/L	248	1 + 3	0.94	0.23 g/L
Titer (normalized)	0–1	83	1 + 3	0.91	0.08
With Spiking	0–3	108	1 + 3	0.97	0.10

## Data Availability

Not applicable.

## Supplementary Materials

The following supporting information can be downloaded at: https://www.mdpi.com/article/10.3390/s22093397/s1, Figure S1: PCA Plot of all spectra used for outlier detection, i.e., points outside Hoteling’s T² ellipse are possible outliers. Colored according to (A) spiking vs. non-spiking, (B) cultivation day, Figure S2: Example Raman spectra taken at three different timepoints during a cultivation. (A) Raw spectra without corrections; (B) Spectra after baseline correction with ALS algorithm—fluorescence underground is removed successfully; (C) Spectra after normalization to the area under the waterband between 1550 and 1750 cm^−1^—removal of intensity differences due to perturbations not caused by the analytes of interest, e.g., by high turbidity in the sample due to high cell counts, Table S1: Overview over spiking solutions and spiking regimen over the duration of the cultivation. Each day from day six onwards samples from 24 Vessels were spiked with shown volumes from one of the two spiking solutions, Table S2: Wavenumber pre-selection for single analytes derived from pre-trials. Several mixtures of the analytes at different concentrations following a DoE approach were prepared. After acquiring a spectrum for each sample, spectra were pre-treated with the ALS-algorithm and separate OPLS for each analyte were built. Very Important Parameters (VIPs), i.e., those Wavenumbers with a high influence on the model were selected and used for future modeling.
