# Supplementary material for: Automated Data Generation for Raman Spectroscopy Calibrations in Multi-Parallel Mini Bioreactors
Source: Sensors (Basel). 2022 Apr 28;22(9):3397. doi: 10.3390/s22093397 (PMC9099804; doi:10.3390/s22093397)
Supplement: Supplementary file 1 [file sensors-22-03397-s001.zip › sensors-1647784-supplementary.pdf]

(A)

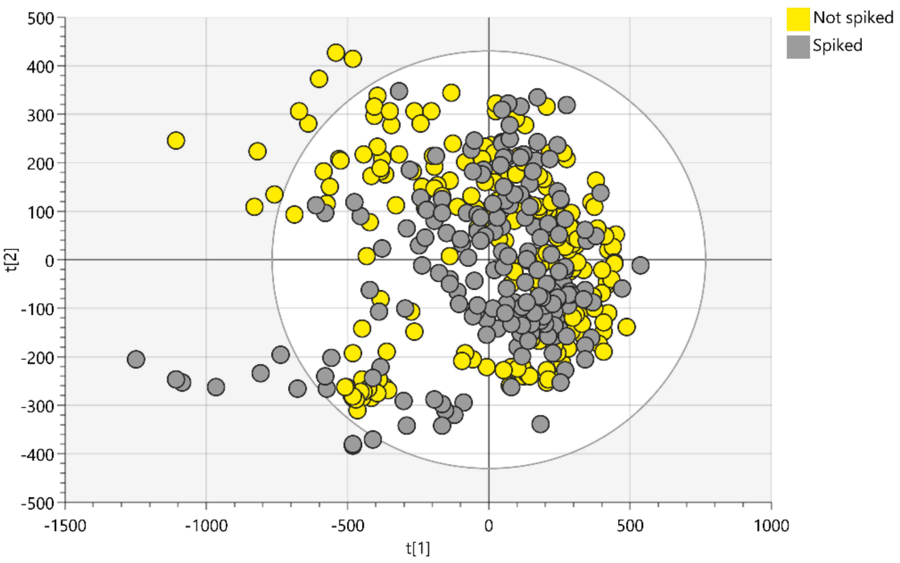

(B)

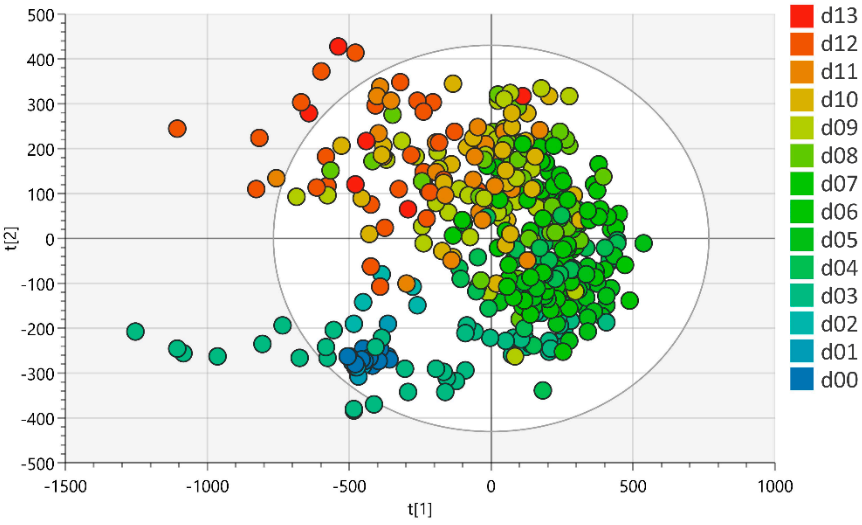

Figure S1: PCA Plot of all spectra used for outlier detection, i.e., points outside Hoteling's T² ellipse are possible outliers. Colored according to (**A**) spiking vs. non-spiking, (**B**) cultivation day.

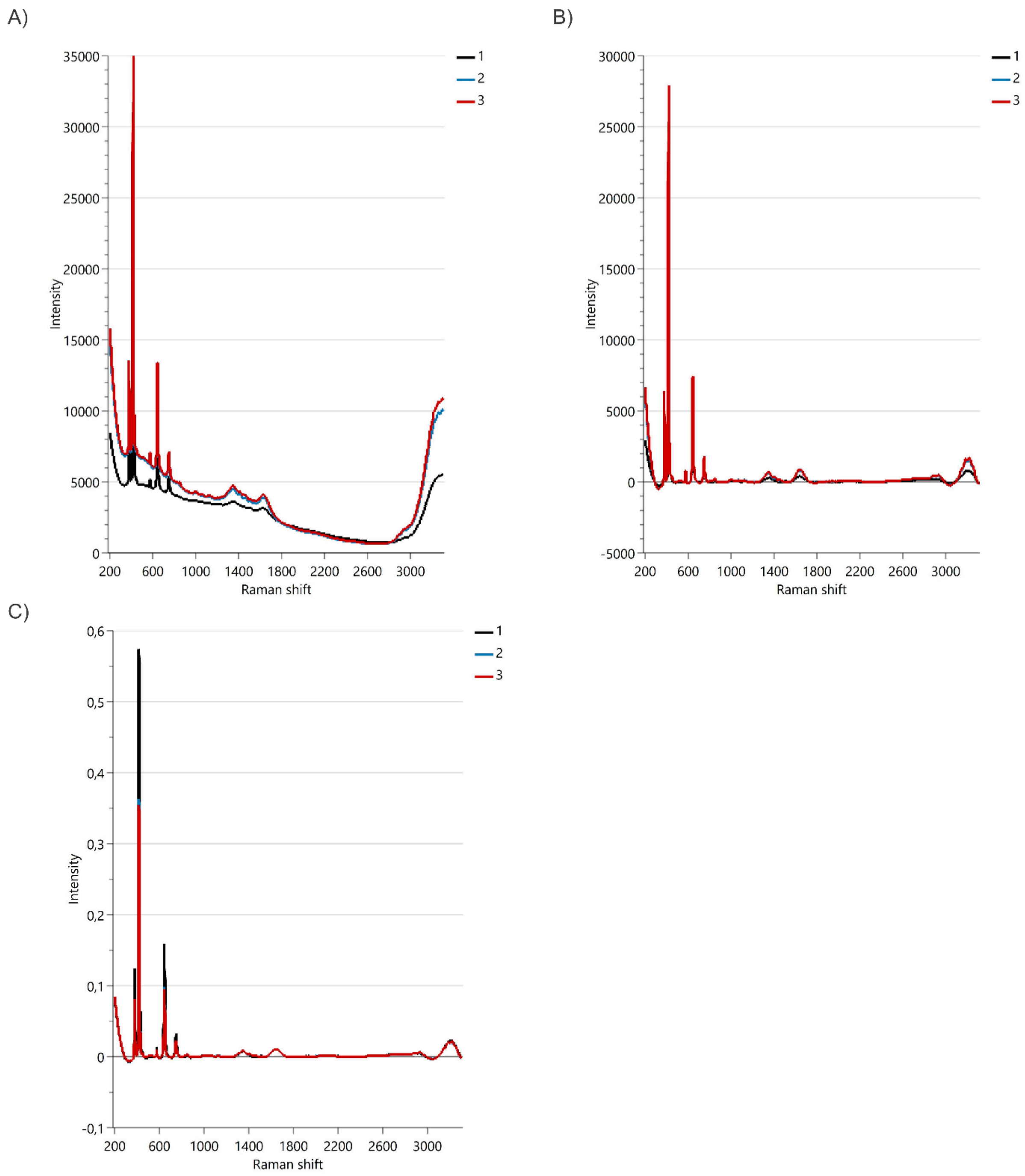

Figure S2: Example Raman spectra taken at three different timepoints during a cultivation. (**A**) Raw spectra without corrections; (**B**) Spectra after baseline correction with ALS algorithm – fluorescence underground is removed successfully; (**C**) Spectra after normalization to the area under the waterband between 1550 and 1750 cm^{-1} – removal of intensity differences due to perturbations not caused by the analytes of interest, e.g., by high turbidity in the sample due to high cell counts.

Table S1: Overview over spiking solutions and spiking regimen over the duration of the cultivation. Each day from day six onwards samples from 24 Vessels were spiked with shown volumes from one of the two spiking solutions.

Analyte	Stock Concentration [g/L]	
	Low	High
Glucose	8	32
Lactate	4	16
Glutamine	4	16
Glutamate	4	16

Day	Spiking Concentration	Volume added
0	-	-
1	-	-
2	-	-
3	-	-
4	-	-
5	-	-
6	High	27
7	High	38
8	Low	48
9	High	48
10	High	57
11	Low	27
12	Low	65
13	High	33

Table S2. Wavenumber pre-selection for single analytes derived from pre-trials. Several mixtures of the analytes at different concentrations following a DoE approach were prepared. After acquiring a spectrum for each sample, spectra were pre-treated with the ALS-algorithm and separate OPLS for each analyte were built. Very Important Parameters (VIPs), i.e., those Wavenumbers with a high influence on the model were selected and used for future modeling.

Glucose	Lactate	Glutamine	Glutamate	Titer
459 - 470	520 - 533	512 - 523	497 - 501	451 - 465
482 - 496	838 - 855	751 - 764	555 - 558	487 - 490
506 - 516	858 - 877	773 - 785	844 - 854	510 - 532
521 - 533	918 - 926	831 - 854	860 - 869	842 - 870
547 - 548	1009 - 1011	858 - 875	885 - 892	904 - 951
837 - 838	1031 - 1038	886 - 905	914 - 934	967 - 969
886 - 897	1047 - 1058	912 - 929	940 - 964	995 - 1031
920 - 935	1071 - 1084	942 - 948	993 - 1001	1051 - 1084
1005 - 1016	1094 - 1109	993 - 997	1007 - 1012	1102 - 1152
1050 - 1061	1116 - 1117	1034 - 1035	1034 - 1037	1170 - 1171
1068 - 1088	1137 - 1138	1068 - 1074	1071 - 1077	1214 - 1215
1097 - 1123	1396 - 1415	1110 - 1116	1088 - 1092	1302 - 1479
1129 - 1150	1422 - 1433	1132 - 1142	1337 - 1345	1605 - 1682
1162 - 1172	1444 - 1452	1163 - 1168	1350 - 1375	
1352 - 1359	1457 - 1468	1283 - 1284	1385 - 1413	
1377 - 1397		1339 - 1348	1419 - 1434	

1452 - 1460		1354 - 1378	1440 - 1443
1467 - 1477		1390 - 1418	1452 - 1476
		1425 - 1436	
		1455 - 1476	
